# A Rare Case of Influenza B Viral Pneumonia in a 10-Day-Old Neonate

**DOI:** 10.7759/cureus.91591

**Published:** 2025-09-04

**Authors:** Noor Ul Ain, Zeeshan Ahmed, Sidra Aman, Musarat Nisa, Arshad Khushdil

**Affiliations:** 1 Department of Pediatrics, Combined Military Hospital, Rawalpindi, PAK; 2 Department of Neonatology, Combined Military Hospital, Rawalpindi, PAK

**Keywords:** atypical pneumonia, influenza b, invasive mechanical ventilation, oseltamivir, term neonate

## Abstract

Influenza B virus (IBV) is known to cause mild to severe respiratory disease in all age groups, but children under five, especially the neonatal age group, are particularly susceptible. IBV is an entity less known to cause disease in neonates. We report a rare case of IBV pneumonia in a 10-day-old neonate who presented to our facility. Baby on arrival was tachypneic and in severe respiratory distress. His suck, grasp, and Moro reflexes were sluggish on initial assessment. He was mechanically ventilated, and supportive respiratory therapy was commenced alongside oseltamivir, to which he responded well. He was discharged successfully after 10 days of hospitalization. Neonatologists should have a high index of suspicion for influenza, especially A/H1N1 and IBV, in neonates reporting with respiratory symptoms in late fall and early spring seasons.

## Introduction

Influenza B virus (IBV) is known to cause mild to severe respiratory disease in all age groups, but children under five are particularly susceptible [1]. Other than being a cause of the seasonal flu epidemics, not much is known about IBV, including the course and impact of disease in the susceptible pediatric age groups, especially the neonates [2]. It is an emerging subclass of the influenza virus.

Guidelines for the administration of the annual influenza vaccine recommend a minimum age of six months at least to be offered the vaccine, with the aim to provide protection to children and impede the community spread of the disease. This vaccine has shown promising results in children, including decreasing the disease duration, overall visits to the hospital, and rates of hospitalization [3]. The influenza vaccine also covers the emerging IBV. While the literature shows promising data on the efficacy of vaccines, especially in children aged 6-59 months [4], to prevent or control seasonal influenza outbreaks, IBV is an entity less known to causes disease in neonates. However, owing to dependency on maternal antibodies and limited immunity in general, neonates are vulnerable to severe IBV infections.

Oseltamivir, the antiviral agent of choice, provides a protective effect of 55%-80% when given after exposure in children. A clinical analysis of influenza viruses in a neonatal intensive care setup in China found Influenza A to be more common than IBV in neonates [5]. Epidemiological data on IBV in neonates are scarce, especially in the lesser developed parts of the world. We, thereby, present a case of IBV pneumonia in a 10-day-old neonate who presented to our facility. Permission was obtained from the parents of the neonate before starting the study.

## Case presentation

We report a case of a 10-day-old boy, born via elective lower section cesarean section at 38 weeks of gestation with a birth weight of 3,500 g. The parents reported to the emergency department of a tertiary care hospital after spending the previous night in a small private care facility because their baby boy had severe breathing difficulty and poor feeding for one day. On arrival to the emergency room (ER), the neonate’s vital signs were as follows: heart rate (HR) 188 beats/min, respiratory rate (RR) 84 breaths/min, peripheral oxygen saturation (SpO₂) 83% on room air, and temperature 98 °F. In the ER, the baby was tachypneic and in severe respiratory distress, with marked subcostal retractions, tracheal tugging, and use of accessory abdominal muscles for breathing. On initial assessment, his suck, grasp, and Moro reflexes were sluggish. No central or acrocyanosis was noted in the baby. Initial management in the ER included supplemental oxygen, with fraction of inspired oxygen (FiO2) maintained at 36-44% (4-6 L/min) via nasal cannula to keep SpO2 above 94%. The neonatology department was contacted for transfer to the neonatal intensive care unit (NICU). The neonatologist assessed the patient in the ER, and he was admitted to and transferred to the NICU.

In the NICU, the parents were briefed about their newborn’s condition. Arterial blood gas analysis revealed acute respiratory acidosis with hypoxemia: pH 7.15 (normal 7.35-7.45), PCO₂ 68 mmHg (normal 35-45 mmHg), PO₂ 55 mmHg (normal 60-80 mmHg), and bicarbonate 22.8 mEq/L (normal 20-24 mEq/L). Because of impending respiratory failure, the baby, who was initially on noninvasive positive pressure ventilation (NIPPV) with minimal improvement, was electively intubated, mechanically ventilated, and sedated. The initial differential diagnosis in this neonate with severe respiratory distress and poor feeding included pneumonia of either bacterial or viral etiology and duct-dependent congenital heart disease (CHD). With these differentials in mind, he was started empirically on intravenous cefotaxime (150 mg/kg/day in three divided doses), intravenous amikacin (15 mg/kg/day once daily), and oral azithromycin (10 mg/kg/day once daily). Intravenous dexamethasone (0.3 mg/kg twice daily) was also administered. Supportive therapy included intravenous fluids with 10% dextrose water and one-fifth normal saline (150 mL/kg/day). The patient was kept nil per os (NPO) initially. Sedation was maintained with an infusion of midazolam (0.5 µg/kg/minute). A complete septic screen, including complete blood count, blood culture, quantitative C-reactive protein (CRP-Q), and serum procalcitonin, was sent alongside a nasopharyngeal swab for respiratory viral studies using multiplex polymerase chain reaction (PCR). Baseline laboratory tests, including liver and renal function panels, were obtained, and an echocardiogram was requested.

The patient responded well to the mechanical ventilation, and his blood gases showed improvement. Clinically, he was vitally stable with no untoward events while being on mechanical ventilation. His echocardiogram showed a small patent foramen ovale (PFO) with a hemodynamically insignificant left-to-right shunt, ruling out a duct-dependent CHD. His blood culture showed no bacterial growth. Serum procalcitonin and CRP-Q were normal, making sepsis less likely. On the second day of admission, IBV was detected on multiplex PCR by the virology department, although the specific strain was not reported. The baby was isolated, and barrier nursing was initiated. The empirical injectable antibiotics were discontinued, and oral oseltamivir (3 mg/kg twice daily), administered orally or via OG tube, was initiated; only syrup azithromycin was continued. Collateral history revealed that the entire family, including a three-year-old sibling, had recovered from a brief flu-like illness the previous week, suggesting possible household transmission to the newborn. Results of serial laboratory tests are presented in Table 1.

**Table 1:** Results of serial laboratory tests done. TLC, total leukocyte count; CRP-Q, quantitative C-reactive protein

Laboratory tests (units)	Day 01	Day 03	Day 07	Reference ranges (for a 10-day-old neonate)
Hemoglobin (g/dL)	15.1	14.7	15.1	10.0-20.0
Hematocrit (%)	47.1	48	47	33-55
TLC (x10^9^/L)	8.5	14.2	12	5.0-20.0
Polymorphonuclear cells (%)	65	64	45	40-60
Lymphocytes (%)	26	18	34	20-40
Platelets (x10^9^/L)	364	287	467	150-450
CRP-Q (mg/L)	1.56	0.61	3.57	<5
Serum procalcitonin (ng/mL)	0.141	0.125	0.05	<0.5
Serum sodium (mmol/L)	143	145	135	135-145
Serum potassium (mmol/L)	4.6	4.2	4.1	3.5-6.0
Serum calcium(mmol/L)	2.27	2.13	2.32	2.0-2.7
Urea (mmol/L)	6.55	5.64	5.93	1.8-5.4
Creatinine (μmol/L)	27	45	35	20-45
Albumin (g/L)	40	39	45	30-50

**Figure 1:**
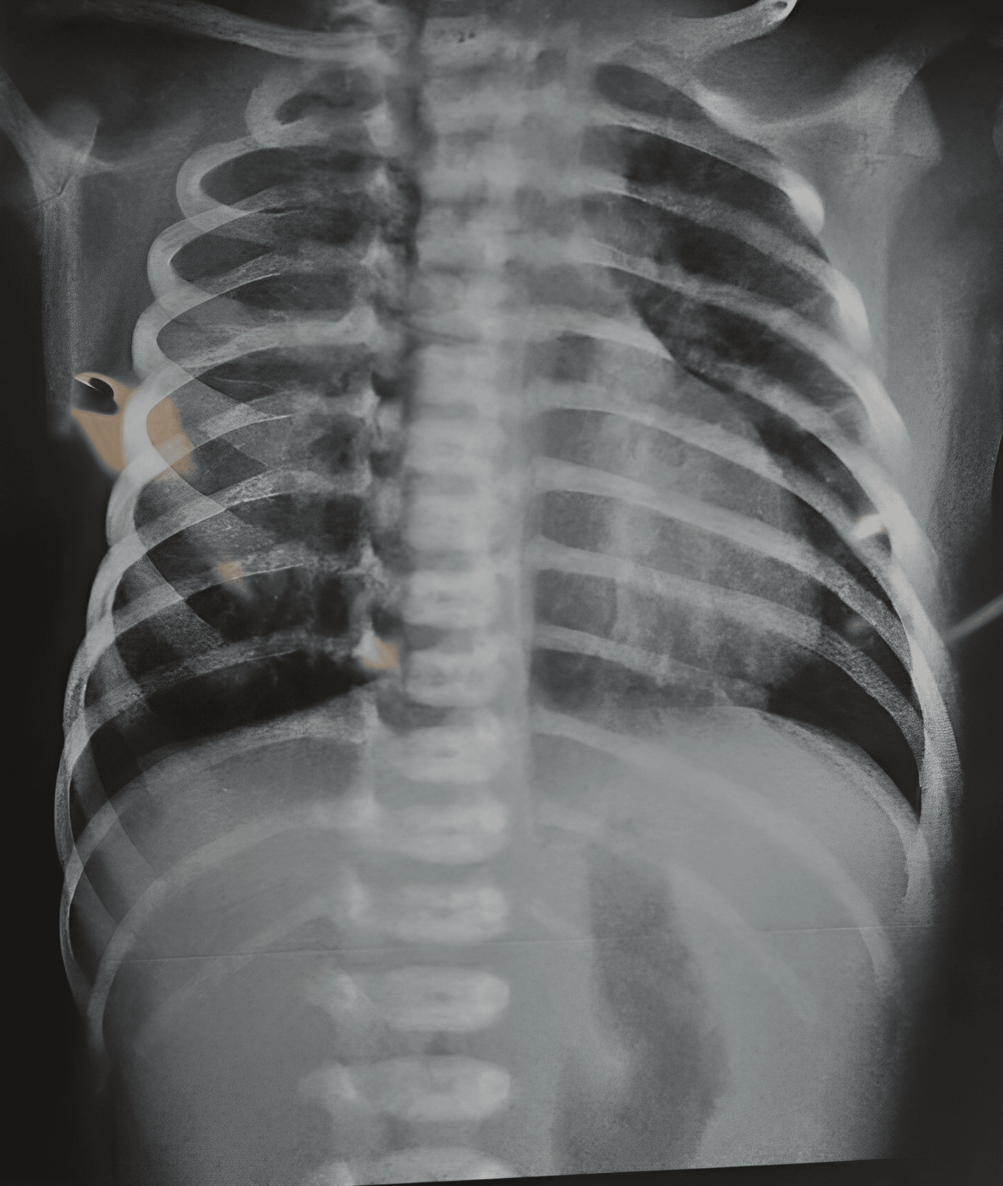
Chest X-ray on admission to the neonatal intensive care unit.

**Figure 2:**
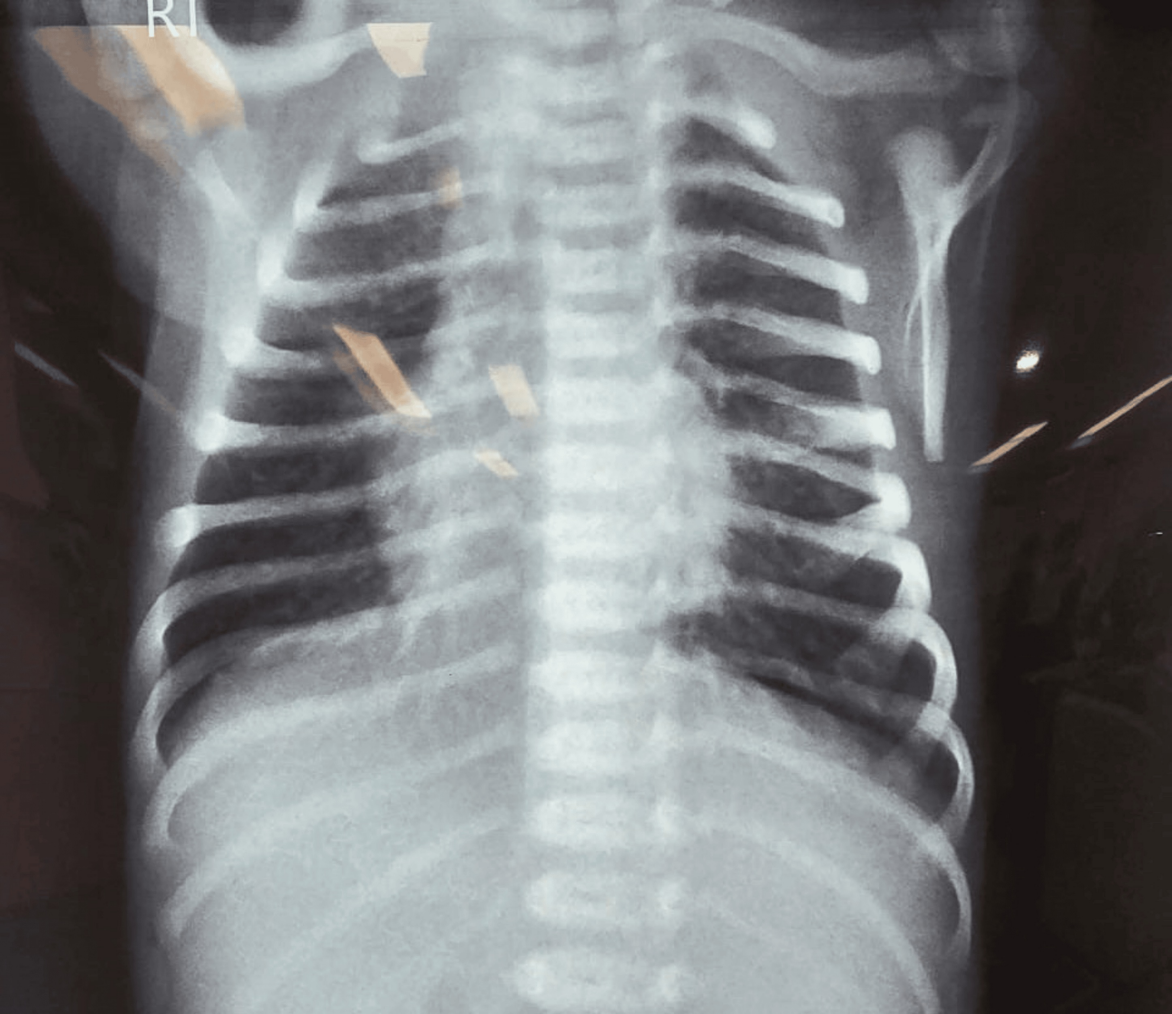
Day 05 chest X-ray of the patient showing resolution of previous lung infiltrates.

On the third day of admission, minimal oral feeds were initiated via orogastric (OG) tube, and a trial to wean him from ventilatory support was attempted. A decision to step up his respiratory therapy was taken with the addition of nebulization using ipratropium and beclomethasone and chest physiotherapy on a six-hourly basis after consultation from a pediatric respiratory physician. The patient was extubated and stepped down to NIPPV, which he tolerated well. Feeding via the OG tube was also gradually increased every day. Chest X-ray on the day of admission is shown in Figure 1. The initial chest radiograph showed scattered right-sided lung involvement, with marked clearing by day 05 (Figure 2), corresponding to clinical improvement in respiratory distress and reduced need for respiratory support.

By day 07, full oral feeds were successfully established, and the neonate was weaned from NIPPV to low-flow nasal oxygen therapy (LFNO). However, due to sudden deterioration and increased work of breathing, he was escalated to bubble continuous positive airway pressure (BCPAP), and oral feeds were temporarily halted. He responded well to the escalation of therapy and remained comfortable afterward. On day 08, he was placed in warmer care and given a trial of oral feeds, initially expressed breast milk, which he tolerated well with a good suck. The patient responded well to the seven-day course of oseltamivir. Oxygen therapy was tapered on day 09, and direct breastfeeding was initiated. He remained for an additional day to address any feeding issues, staying in mother-based care with vital sign monitoring. After maintaining steady weight gain over the last three inpatient days, he was discharged with advice to follow up with a neonatologist and a pediatric pulmonologist on an outpatient basis. In summary, the patient, a 10-day-old neonate presenting with severe respiratory distress in the pediatric ER, was admitted to the NICU with suspected severe pneumonia or possible duct-dependent CHD. He was initially started on empirical antibiotics and intravenous steroids but was eventually switched to syrup oseltamivir after multiplex PCR detected IBV. He responded well to respiratory therapy and was discharged after a 10-day inpatient stay.

## Discussion

The global incidence of influenza in children under five years is estimated at 90 million cases per year [6]. The annual recurrence of influenza in the form of epidemics is mainly attributed to the immune escape and resurfacing of new variants owing to mutations and reassortments [7]. While vaccines and post-exposure prophylaxis are reliable means to interrupt the spread in the community, the same cannot be generalized for neonates, who are at greatest risk for severe respiratory disease caused by influenza viruses, owing to their limited immunity and difficulty acclimatizing to the new environment.

IBV has been classified into two distinct antigenic strains, namely, strain B/Victoria/2/87, the most recent reference strain, or B/Yamagata/16/88, a variant that was isolated in Japan in May 1988 [8]. The mode of transmission is mainly through respiratory droplet inhalation, and the virus, being very contagious, spreads rapidly from one person to another. A case series in Germany reported six very low birth weight (VLBW) neonates with influenza A/H1N1 infection, with one fatal case having systemic inflammatory response syndrome, establishing the possibility of fatality secondary to perinatal transmission [9]. 

A case reported in Israel described influenza A/H1N1 (swine flu) infection in a 50-day-old very low birth weight (VLBW) premature infant, who was successfully treated with intermittent NIPPV and supportive therapy. Reduced levels of maternally transferred passive immunity were identified as one factor contributing to susceptibility in such neonates [10]. Indeed, influenza virus, especially IBV, a lesser-known disease entity in neonates, can, under circumstances similar to those in our case, affect neonatal units, potentially impacting staff, including pregnant women, or other unimmunized susceptible individuals. So, adequate safety precautions for nursing such patients need to be taken. Neonatologists should also have a high index of suspicion for influenza, especially IBV, in neonates reporting with respiratory symptoms in late fall and early spring seasons.

## Conclusions

IBV is known to cause mild to severe respiratory disease in all pediatric age groups, but children under 5, especially the neonates, are particularly susceptible. While vaccines and post-exposure prophylaxis interrupt the community spread of the disease, the same cannot be generalized for neonates, who are at greatest risk for severe disease by influenza viruses, owing to their limited immunity. Suspecting influenza infection in neonates and early treatment with antiviral and supportive therapy result in good clinical outcomes. There is a dire need, however, to conduct elaborate studies regarding influenza viral infections in neonates.
